# Two cases of breast carcinoma with osteoclastic giant cells: Are the osteoclastic giant cells pro-tumoural differentiation of macrophages?

**DOI:** 10.1186/1746-1596-5-55

**Published:** 2010-08-23

**Authors:** Yukiko Shishido-Hara, Atsushi Kurata, Masachika Fujiwara, Hiroki Itoh, Shigeru Imoto, Hiroshi Kamma

**Affiliations:** 1 Department of Pathology, Kyorin University School of Medicine 6-20-2, Shinkawa, Mitaka, Tokyo 181-8611, Japan; 2 Department of Breast Surgery, Kyorin University School of Medicine 6-20-2, Shinkawa, Mitaka, Tokyo 181-8611, Japan

## Abstract

Breast carcinoma with osteoclastic giant cells (OGCs) is characterized by multinucleated OGCs, and usually displays inflammatory hypervascular stroma. OGCs may derive from tumor-associated macrophages, but their nature remains controversial. We report two cases, in which OGCs appear in common microenvironment despite different tumoural histology. A 44-year-old woman (Case 1) had OGCs accompanying invasive ductal carcinoma, and an 83-year-old woman (Case 2) with carcinosarcoma. Immunohistochemically, in both cases, tumoural and non-tumoural cells strongly expressed VEGF and MMP12, which promote macrophage migration and angiogenesis. The Chalkley count on CD-31-stained sections revealed elevated angiogenesis in both cases. The OGCs expressed bone-osteoclast markers (MMP9, TRAP, cathepsin K) and a histiocyte marker (CD68), but not an MHC class II antigen, HLA-DR. The results indicate a pathogenesis: regardless of tumoural histology, OGCs derive from macrophages, likely in response to hypervascular microenvironments with secretion of common cytokines. The OGCs have acquired bone-osteoclast-like characteristics, but lost antigen presentation abilities as an anti-cancer defense. Appearance of OGCs may not be anti-tumoural immunological reactions, but rather pro-tumoural differentiation of macrophage responding to hypervascular microenvironments induced by breast cancer.

## Background

Breast carcinoma with osteoclastic giant cells is a rare entity that falls under the WHO classification of invasive ductal carcinoma, not otherwise specified [1]. This tumor is characterized by the presence of osteoclastic giant cells (OGCs), the nature of which remains controversial. OGCs accompany a variety of breast tumors, including invasive ductal carcinoma and cribriform, tubular, mucinous, papillary, lobular, squamous, and other metaplastic patterns. Despite divergent tumor histology, most cases present a well-demarcated mass with characteristic inflammatory and hypervascular stroma [2].

OGCs are agreed to be of histiocytic origin, and are hypothesized to derive from macrophages [3]. Macrophages display marked plasticity with both pro-tumoural and anti-tumoural activities [4-6]. Their classical anti-tumoural roles include promotion of specific immunity by inducing T cell activation via antigen presentation. Recent studies have also focused on their direct or indirect pro-tumoural functions: enhancement of angiogenesis and cancer cell growth and spread [4,6]. Macrophages secrete growth factors such as epidermal growth factor (EGF) and vascular endothelial growth factor (VEGF), and produce proteases including matrix metalloproteinases (MMPs). Both VEGF and MMP12 enhance macrophage migration [7,8], and VEGF also regulates angiogenesis and lymphangiogenesis through different types of receptors. Microenvironments, with secretion of cytokines, seem to affect progression of breast cancer [9,10], and may also determine whether OGCs are formed. However, in current thinking, the prognosis of breast carcinoma with OGCs is considered to be related to the tumoural histology, and not influenced by the presence of OGCs [1].

Here, we report two cases of breast carcinoma with OGCs associated with invasive ductal carcinoma (Case 1) or carcinosarcoma (Case 2). Despite different tumoural histology, two cases displayed common microenvironments with expression of VEGF and MMP12, suggesting enhanced macrophage migration and angiogenesis. The OGCs presented phenotypic resemblance to the osteoclasts in the bone, and lacked antigen presentation abilities. Macrophage plasticity responding microenvironments is discussed, in relation to prognosis of breast carcinoma.

## Case Presentation

Case 1: A 44-year-old woman presented with a lump in the lower inner quadrant of her right breast. Physical examination revealed a well-demarcated firm tumor with good mobility. Mammography and ultrasonography revealed a well-circumscribed tumor of 30 × 20 × 25 mm, and magnetic resonance imaging (MRI) showed rich vascularity, especially in the periphery. Fine-needle aspiration and core needle biopsy demonstrated invasive ductal carcinoma with multi-nucleated OGCs. Partial mastectomy was performed following sentinel lymph node biopsy. There was no metastasis to the sentinel lymph nodes, and the postoperative stage was pT2 N0 M0, stage IIA.

Case 2: An 83-year-old woman presented with a painful lump in the upper inner quadrant of her right breast. Ultrasonography revealed a well-defined mass of 19 × 16 × 10 mm. Both mammography and MRI suggested malignancy. As the specimen of aspiration cytology did not contain enough epithelial cells for diagnosis, an intraoperative frozen section was examined, leading to diagnosis of malignant tumor. Partial mastectomy was performed, and the final pathologic diagnosis was beast carcinoma with OGCs. The postoperative stage for this patient was also pT2 N0 M0, stage IIA.

## Materials and methods

For histological analysis, the surgical specimens were fixed in 10% buffered formalin, embedded in paraffin, sectioned, and stained with hematoxylin and eosin. For immunohistochemical analysis, the sections were deparafinized and reacted with primary antibodies, followed by the immunoperoxidase method with a commercial kit (DakoCytomation Co Ltd, Glostrup, Denmark). The primary antibodies used in this study are as follows: ER (1D5, 1:100, Dako), PgR (PgR636, 1:400, Dako), HER2 (polyclonal, 1:1, Dako), CKAE1/AE3 (AE1/AE3, 1:100, Dako), Vimentin (V9, 1:2, Nichirei), VEGF (A-20, 1:400, Santacruz), MMP9 (polyclonal, 1:5000, Abcam), MMP12 (polyclonal, 1:100, Abcam), CD31 (JC70A, 1:50, Dako), CD68 (PGM-1, 1:100, Dako), HLA-DR (TAL1B5, 1:100, Dako), TRAP (26E5, 1:100, Novocastra), Cathepsin K (182-12G5, 1:10000, Dai-ichi fain chemical).

## Results

### Gross and microscopic findings

**Figure 1:**
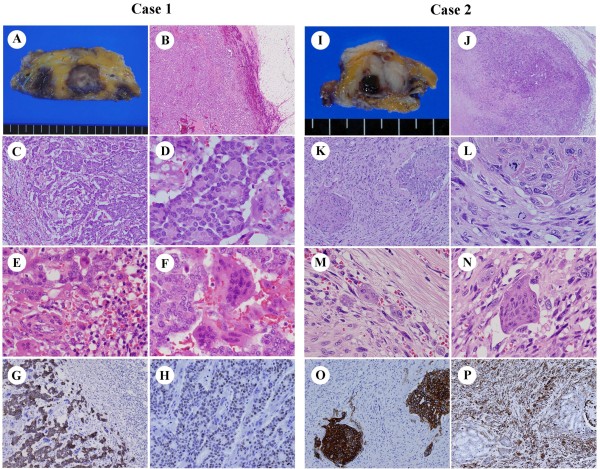
**Gross and microscopic findings of breast carcinoma with OGCs**. OGCs in Case 1 appeared associated with invasive ductal carcinoma, grade 1, and in Case 2 with carcinosarcoma. **A-H**: Case 1. **A**: Gross appearance. **B**: Low power view of the tumor. **C, D**: High power views of the tumor. **E, F**: Bi-, tri-, tetranucleate cells were observed as well as OGCs in hypervascular inflammatory stroma. **G**: CK AE1/AE3 staining. **H**: ER staining. **I-N**: Case 2, corresponding to A-F in Case 1. **O**: CK AE1/AE3 staining. **P**: Vimentin staining.

Case 1 grossly showed a well-circumscribed solid tumor, measuring 3.5 × 2.5 cm on the maximum cut plane. The tumor appeared gray to white in the center, but hemorrhagic dark brown at the periphery (Figure 1A). Microscopically, the tumor was surrounded by hyperemic blood vessels (Figure 1B). The ductal carcinoma cells, which had relatively small and round nuclei with mild atypia and infrequent mitosis, formed distinct glandular and cribriform structures, equivalent to invasive ductal carcinoma, grade 1 (well-differentiated) (Figure 1C, D). The hypervascular stroma contained numerous inflammatory cells and multinucleated giant cells (Figure 1E). Some giant cells showed a polarized cell body resembling activated osteoclasts (Figure 1F). Immunohistochemically, strong staining for cytokeratin (CK) AE1/AE3 was observed in all tumor cells, but not in OGCs (Figure 1G). About 40% of tumor cells were positive for estrogen receptor (ER) (Figure 1H) and progesterone receptor (PgR), but negative for HER2, phenotypically corresponding to Luminal A type. Following the WHO classification, the diagnosis was breast carcinoma with OGCs.

The tumor in Case 2 also showed a well-circumscribed gross appearance, measuring 2.2 × 1.5 cm on the maximum cut plane. The tumor was rather whitish, but contained a small (5 mm) hemorrhagic area (Figure 1I). Microscopically, the main part of tumor consisted of spindle-shaped sarcomatous cells with frequent mitotic figures, and there were also foci of ductal carcinoma cells with an intraductal component (Figure 1J-L). Inflammatory cells and multinucleated OGCs were seen in the hypervascular stroma (Figure 1M, N). Immunohistochemically, the ductal carcinoma cells were positive for CK AE1/AE3 (Figure 1O) but not ER, PgR, or HER2. The spindle-shaped tumor cells constituting most of the tumor were not reactive for CK AE1/AE3, but instead were strongly positive for vimentin (Figure 1P). Thus, the case was diagnosed as breast carcinoma with OGCs, and the tumor was equivalent to metaplastic carcinoma with mesenchymal component, corresponding to so-called carcinosarcoma.

### Topography of CD68-positive cells and expression of chemotactic agents

**Figure 2:**
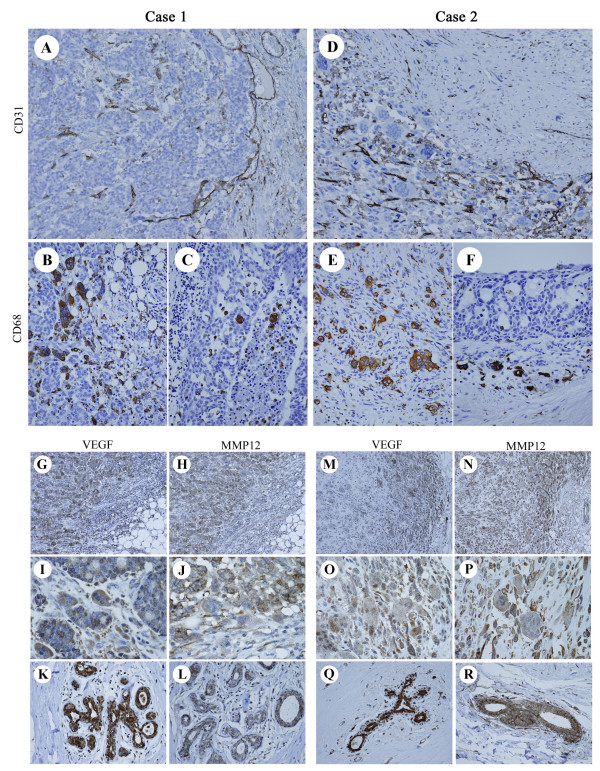
**Topography of CD68-positive cells and expression of VEGF and MMP12**. Case 1 and Case 2 showed distinct vascular patterns, but in both cases OGCs preferentially appeared in hypervascular stroma. Marked expression of VEGF and MMP12 was evident. **A**: Distribution of blood vessels marked with CD31 in Case 1. **B, C**: CD68-positive cells in Case 1. **D**: Distribution of blood vessels marked with CD31 in Case 2. **E, F**: CD68-positive cells in Case 2. **G-R**: VEGF and MMP12 expression in Case 1 (G-L), and Case 2 (M-R).

Despite the different histological features of the breast carcinoma cells, the OGCs in both cases showed similar morphology, and large OGCs contained 20-30 nuclei. In both cases, OGCs preferentially appeared in hypervascular stroma, but the topography of OGCs and blood vessels differed between Case 1 and Case 2. In Case 1, CD31 immunostaining demonstrated that the smaller blood vessels were relatively evenly distributed within the tumor; in contrast, numerous enlarged blood vessels were seen in the periphery (Figure 2A). Large OGCs with over 20 nuclei showed concentric topography; they were mainly seen in the central invasive lesion, while mono-, bi-, tri-, or oligonucleate CD68-positive cells were usually observed in the periphery (Figure 2B). Typical OGCs containing more than 20 nuclei were scarcely detected in the intraductal components, although some CD68-positive cells, including mono-, bi- or trinucleate cells, were seen within the ducts (Figure 2C). In Case 2, unlike Case 1, the distribution of OGCs and blood vessels was irregular, not concentric. OGCs were usually clustered in irregularly distributed hypervascular areas (Figure 2D). Mono-, bi-, tri-, or oligonucleated cells were also observed around the OGCs (Figure 2E). However, CD68-positive cells, regardless of the number of the nuclei, were scarcely seen within the minor intraductal lesions (Figure 2F).

To assess the microenvironment of the tumors, we examined the expression of two chemoattractants for macrophage migration and angiogenesis, VEGF and MMP12. Prominent expression of VEGF and MMP12 was observed in most tumor cells, most inflammatory cells, and even neighboring normal mammary glands both in Case 1 and Case 2 (Figure 2G-R). The OGCs, in both cases, also displayed marked expression of VEGF and MMP12.

### Statistic evaluation of microvessel density

In hypervascular stroma of both cases, microvessel density was evaluated according to the Chalkley count on CD-31-stained sections, as described earlier. The mean values of the three most vascular areas (hot spot) were 9.6 for Case 1 and 10.7 for Case 2, respectively. These counts were much higher than the average Chalkley count of a total 330 invasive breast carcinoma cases, 5.75 (range 2.33 - 10.67, median 5.67, SD 1.54), reported in a previous study[11,12].

### Phenotype of the OGCs in the breast

**Figure 3:**
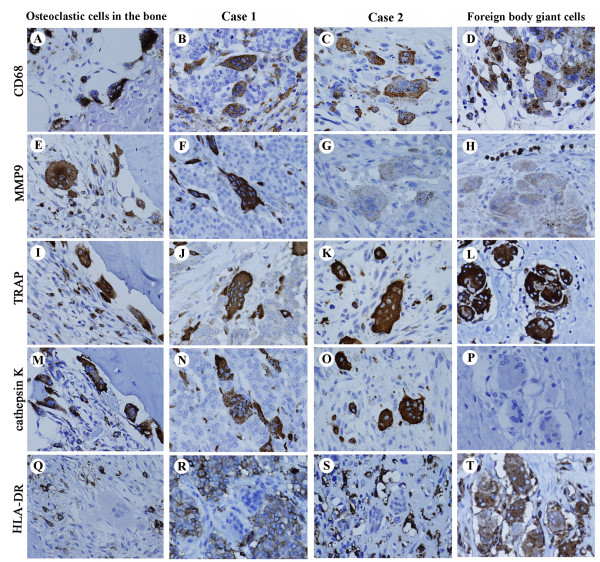
**Expression of phenotypic markers of OGCs**. Phenotypes of OGCs in the breast (center panels) were compared those of osteoclastic cells in the bone (far left) and foreign body giant cells (far right). OGCs in the breast displayed both osteoclastic and histiocytic characteristics but lacked antigen presentation abilities. **A-D**: CD68, a phenotypic marker for histiocytes. **E-H**: MMP9, a broad marker for macrophage-osteoclast lineage cells. **I-L**: TRAP. **M-P**: Cathepsin K. **Q-T**: HLA-DR.

Phenotypic characteristics of OGCs in the two cases were evaluated and compared with those of osteoclastic cells in fibrous dysplasia of the rib bone (from a 45-year-old woman) and foreign-body giant cells in re-operated breast tissue (a 42-year-old woman). CD68, a histiocytic marker, was detected on all macrophage lineage cells, including OGCs and oligonucleated giant cells in both Case 1 and Case 2, osteoclastic cells in the bone sample, and foreign-body giant cells (Figure 3A-D). MMP9 is a broad marker for macrophage-osteoclast lineage cells, including mononuclear precursors, fused polykaryons, and mature osteoclasts in bone. MMP9 expression was distinctly detected in the osteoclastic cells (Figure 3E) and the OGCs in Case 1 (Figure 3F), but markedly weaker in Case 2 (Figure 3G) and foreign-body giant cells (Figure 3H). TRAP and cathepsin K, lytic enzymes for bone resorption, are functional markers for osteoclast-lineage cells. Their expression was distinct in the osteoclastic cells and the OGCs in both Case 1 and Case 2 (Figure 3I-K, M-O). Unexpectedly, foreign body giant cells were also strongly positive for TRAP (Figure 3L), although they were negative for cathepsin K (Figure 3P). HLA-DR, an MHC class II antigen, is generally expressed in antigen-presenting cells including macrophages and peripheral blood mononuclear cells. Only foreign body giant cells were positive for HLA-DR; the bone osteoclasts and OGCs were negative (Figure 3Q-T).

## Discussion

In this study, we demonstrated that the OGCs appear in relation to inflammatory hypervascular stroma around breast carcinoma regardless of histology. Abundant VEGF and MMP12 were secreted from both tumoural and non-tumoural cells, and these cytokines promote macrophage migration and angiogenesis. Notable increase of microvessel density was actually shown by the Chalkley count in inflammatory stroma of both cases. The OGCs are likely generated by syncytial fusion of macrophages, but not by mitosis without cell division. Thus, bi-, tri-, or oligo-nuclear CD-68 positive cells were scattered around OGCs, but nuclear mitotic figures were not observed. Phenotypic resemblance of OGCs to the osteoclasts in the bone was confirmed with expression of MMP9, TRAP, and cathepsin K. The OGCs were negative for HLA-DR, and lacked antigen presentation abilities as anti-cancer defence.

Macrophages are multifunctional, showing marked phenotypic plasticity in response to microenvironments. Indeed, tumor-associated macrophages (TAMs) isolated from breast cancer *in vitro *differentiated into multinucleated giant cells with bone resorption [3]. A characteristic inflammatory and hypervascular stroma is commonly observed in breast carcinoma with OGCs, regardless of histology of tumoural cells. This characteristic stroma may indicate secretion of specific cytokines, and we, in this study, first demonstrated marked expression of VEGF and MMP12. Therefore, appearance of OGCs may not be anti-tumoural immunological reactions, but rather pro-tumoural differentiation of macrophage responding to hypervascular microenvironments induced by breast cancer. However, the further case series study is necessary to elucidate the prognostic or biological significance of OGCs in association with breast carcinoma.

In the bone tissue, osteoclast differentiation seems tightly associated with signaling related to RANKL (receptor activator of nuclear factor-κB ligand) [13], or VEGF and its receptor Flt-1 [14]. Flt-1, expressed on human monocyte/macrophage lineage cells [15], also regulates macrophage migration in response to VEGF [7]. To test for such differentiation signaling in the OGCs in the breast, we also examined expression of RANKL and Flt-1. Weak but distinct expression of Flt-1 was detected in the OGCs of both Case 1 and Case 2, but expression of RANKL was not clear (data not shown). Thus, it is still uncertain if osteoclast differentiation signaling function in the breast, mimicking in the bone.

## Conclusion

The OGCs likely develop from macrophages in response to pro-tumoural microenvironment defied by cytokines, favoring macrophage migration and angiogenesis. The OGCs have acquired bone-osteoclast-like characteristics, but no more have antigen presentation abilities as anti-cancer defence. Macrophages and angiogenesis may imply the poor prognosis of the breast cancer [16,17]. Therefore, the appearance of OGCs is not merely histiocytic reactions, but better taken as a part of pathology of breast tumours.

## Consent

Written informed consent was obtained from both patients for publication of this case report and any accompanying images.

## Competing interests

The authors declare that they have no competing interests.

## Authors' contributions

YS-H, AK, MF and HK participated in conception of the idea and writing of the manuscript. HI and SH provided the clinical data and edited the clinical case presentation. All authors read and approved the final manuscript.

## References

[B1] Fattaneh A Tavassolo B Devilee P World Health Organization Classification of Tumours. Pathology and Genetics of Tumor of the breast and female genital organs 2003 Lyon, IARC Press

[B2] Agnanits NT Rosen PP Rosen's breast pathology 2001 Philadelphia, Lippincot Williams & Wilkins

[B3] Quinn JM McGee JO Athanasou NA Human tumour-associated macrophages differentiate into osteoclastic bone-resorbing cells J Pathol 1998 184 31 36 10.1002/(SICI)1096-9896(199801)184:1<31::AID-PATH962>3.0.CO;2-V 9582524

[B4] Lewis CE Pollard JW Distinct role of macrophages in different tumor microenvironments Cancer Res 2006 66 605 612 10.1158/0008-5472.CAN-05-4005 16423985

[B5] Mosser DM Edwards JP Exploring the full spectrum of macrophage activation Nat Rev Immunol 2008 8 958 969 10.1038/nri2448 19029990 PMC2724991

[B6] Siveen KS Kuttan G Role of macrophages in tumour progression Immunol Lett 2009 123 97 102 10.1016/j.imlet.2009.02.011 19428556

[B7] Barleon B Sozzani S Zhou D Weich HA Mantovani A Marme D Migration of human monocytes in response to vascular endothelial growth factor (VEGF) is mediated via the VEGF receptor flt-1 Blood 1996 87 3336 3343 8605350

[B8] Nenan S Boichot E Lagente V Bertrand CP Macrophage elastase (MMP-12): a pro-inflammatory mediator? Mem Inst Oswaldo Cruz 2005 100 Suppl 1 167 172 15962117 10.1590/s0074-02762005000900028

[B9] Pollard JW Macrophages define the invasive microenvironment in breast cancer J Leukoc Biol 2008 84 623 630 10.1189/jlb.1107762 18467655 PMC2516896

[B10] Yu JL Rak JW Host microenvironment in breast cancer development: inflammatory and immune cells in tumour angiogenesis and arteriogenesis Breast Cancer Res 2003 5 83 88 10.1186/bcr573 12631386 PMC154151

[B11] Dhakal HP Bassarova A Naume B Synnestvedt M Borgen E Kaaresen R Schlichting E Wiedswang G Giercksky KE Nesland JM Breast carcinoma vascularity: a comparison of manual microvessel count and Chalkley count J Pathol 1998 184 31 36 10.1002/(SICI)1096-9896(199801)184:1<31::AID-PATH962>3.0.CO;2-V 19554512

[B12] Hansen S Sørensen FB Vach W Grabau DA Bak M Rose C Microvessel density compared with the Chalkley count in a prognostic study of angiogenesis in breast cancer patients Histopathology 2004 44 428 36 10.1111/j.1365-2559.2004.01848.x 15139990

[B13] Boyle WJ Simonet WS Lacey DL Osteoclast differentiation and activation Nature 2003 423 337 342 10.1038/nature01658 12748652

[B14] Aldridge SE Lennard TW Williams JR Birch MA Vascular endothelial growth factor receptors in osteoclast differentiation and function Biochem Biophys Res Commun 2005 335 793 798 10.1016/j.bbrc.2005.07.145 16105658

[B15] Sawano A Iwai S Sakurai Y Ito M Shitara K Nakahata T Shibuya M Flt-1, vascular endothelial growth factor receptor 1, is a novel cell surface marker for the lineage of monocyte-macrophages in humans Blood 2001 97 785 791 10.1182/blood.V97.3.785 11157498

[B16] Leek RD Hunt NC Landers RJ Lewis CE Royds JA Harris AL Macrophage infiltration is associated with VEGF and EGFR expression in breast cancer J Pathol 2000 190 430 436 10.1002/(SICI)1096-9896(200003)190:4<430::AID-PATH538>3.0.CO;2-6 10699991

[B17] Leek RD Harris AL Tumor-associated macrophages in breast cancer J Mammary Gland Biol Neoplasia 2002 7 177 189 10.1023/A:1020304003704 12463738

